# Comparison of two olfactory tests in young children: A study of crayon sniffing (C-Sniff) and short U-Sniff (qU-Sniff)

**DOI:** 10.1007/s00405-026-10166-3

**Published:** 2026-03-23

**Authors:** Janine Gellrich, Alicia Jatzek, Claudia Galvao, Valentin A. Schriever, Thomas Hummel

**Affiliations:** 1 Abteilung Neuropädiatrie, Department of Pediatrics, Faculty of Medicine, University Hospital Carl Gustav Carus, Technische Universität Dresden, Fetscherstrasse 74, Dresden, 01307 Germany; 2 Department of Otorhinolaryngology, Smell & Taste Clinic, Technical University of Dresden, Dresden, Germany; 3 NoAr Brasil S.A, Sao Paulo, Brazil; 4 Department of Pediatrics, Division of Pediatric Neurology, Neurometabolics and Prevention, Goethe University Frankfurt, Frankfurt (Main), Germany

**Keywords:** Olfaction, Toddler, Olfactory testing, Odor identification

## Abstract

**Aims:**

This study aimed to evaluate and compare the feasibility and reliability of two olfactory tests for toddlers aged 30 to 48 months: the established short Universal Sniff (qU-Sniff) and the novel Crayon-Sniff (C-Sniff).

**Methods:**

The study included 168 toddlers aged 30 to 48 months. A primary analysis comparing the two tests regarding feasibility (completion time, dropout rates) and the influence of potential factors (age, gender, test sequence) was conducted on all participants (*N* = 168). The sequence of the two tests was randomized to control for order effects. Additionally, test-retest reliability was assessed in a subgroup of 56 children (the oldest age group), resulting in 224 total measurement sets for the study.

**Results:**

Mean identification scores were comparable between the qU-Sniff (M = 1.75) and the C-Sniff (M = 1.88), *p* = 0.8. The C-Sniff required a significantly longer completion time (mean 352 s) than the qU-Sniff (mean 161 s, *p* < 0.0001). While overall dropout rates did not differ significantly between tests, younger children (30–36 months) exhibited significantly higher dropout rates for both the qU-Sniff and C-Sniff compared to older children. The order in which the tests were administered had a significant effect on completion times, but not on test scores or dropout rates. Both the qU-Sniff (ICC = 0.52) and C-Sniff (ICC = 0.539) demonstrated moderate test-retest reliability. There was no significant difference in scores between genders, and the amount of time spent in daycare had no significant effect on test performance.

**Conclusion:**

Both the qU-Sniff and C-Sniff are moderately reliable tools for assessing olfaction in toddlers. However, the C-Sniff is significantly more time-consuming. Future studies should consider the age-related challenges and the significant sequence effects on test duration.

## Introduction

The odor identification ability of humans follows a characteristic pattern over the lifespan: it rises in childhood up to a maximum in adolescence and early adulthood and decreases in older age [1]. From this observation, a mythological question arises: Does the lower odor identification ability in early childhood really display a lower olfactory ability, or is it a consequence of our testing method? The testing of olfaction requires, besides an olfactory function, an ability to concentrate, compliance, and understanding of complex instructions. Therefore, it is crucial to develop olfactory tests that lower the age boundaries depending on executive function, verbal abilities, and compliance [2, 3] to measure olfactory function reliably.

The ability to smell is of importance for the development of children, because it plays a major role in nutrition and the avoidance of potential household accidents or food poisoning [4]. The early and precise detection of olfactory dysfunction is of relatively high clinical interest because it could be an indicator of different neurological diseases or developmental delays. As a reaction to these boundaries in recent years, different approaches have been made to develop child-friendly olfactory tests for school-aged children [3]. Nevertheless, there is still a lag in knowledge in testing children at the age of a toddler, especially between the ages of 30 and 48 months. In this young cohort, the boundaries of test compliance, acceptance, and distraction tolerance are especially high. The presented study aimed to close this gap in knowledge, as we compare two methods for odor identification in this age cohort: the already presented short U-Sniff (qU-Sniff) and the new Crayon-Sniff (C-Sniff).

The qU-Sniff, a short version of the U-Sniff, uses Sniffin’ Sticks, which spread their odor by removing the cap and being held right under the nose. The abstract and passive presentation could reduce the acceptance and compliance in toddlers.

As an alternative, the C-Sniff has been developed, which uses the same five odors as the qU-Sniff in crayons instead of Sniffin’ Sticks. The children are asked to draw with one crayon at a time and then to sniff the paper to identify the odor of the crayon. Through implementing the odor presentation in a familiar and playful activity, the goal was to improve the motivation, compliance, and acceptance of the testing. This method is expected to get the toddlers more involved in the testing.

This prospective randomized study aimed to evaluate and compare the feasibility, acceptability, and test-retest reliability of the new C-Sniff and the qU-Sniff in 168 toddlers aged 30 to 48 months.

## Materials and methods

The study was designed as a prospective, randomized trial to compare two olfactory testing methods in young children. The study was registered in “Deutsches Studienregister” (DRKS00038269) and received the approval of the local ethics committee (BO-EK-492112020). Participants were recruited from daycare centers. Inclusion criteria were kindergarten children aged 30 to 48 months who, according to their parents, were healthy, developing normally, and had no known olfactory disorders. Exclusion criteria were a lack of parental consent, language development delay, or chronic illness.

A total of 168 children were included in the main study cohort. They were separated into three equally sized age groups (*n* = 56 each): 30–36 months, 37–42 months, and 43–48 months. Participants were also evenly separated by sex (male/female). The children in the oldest age group (3, *n* = 56) were designated as the retest cohort and examined a second time to determine test-retest reliability.

**Fig. 1:**
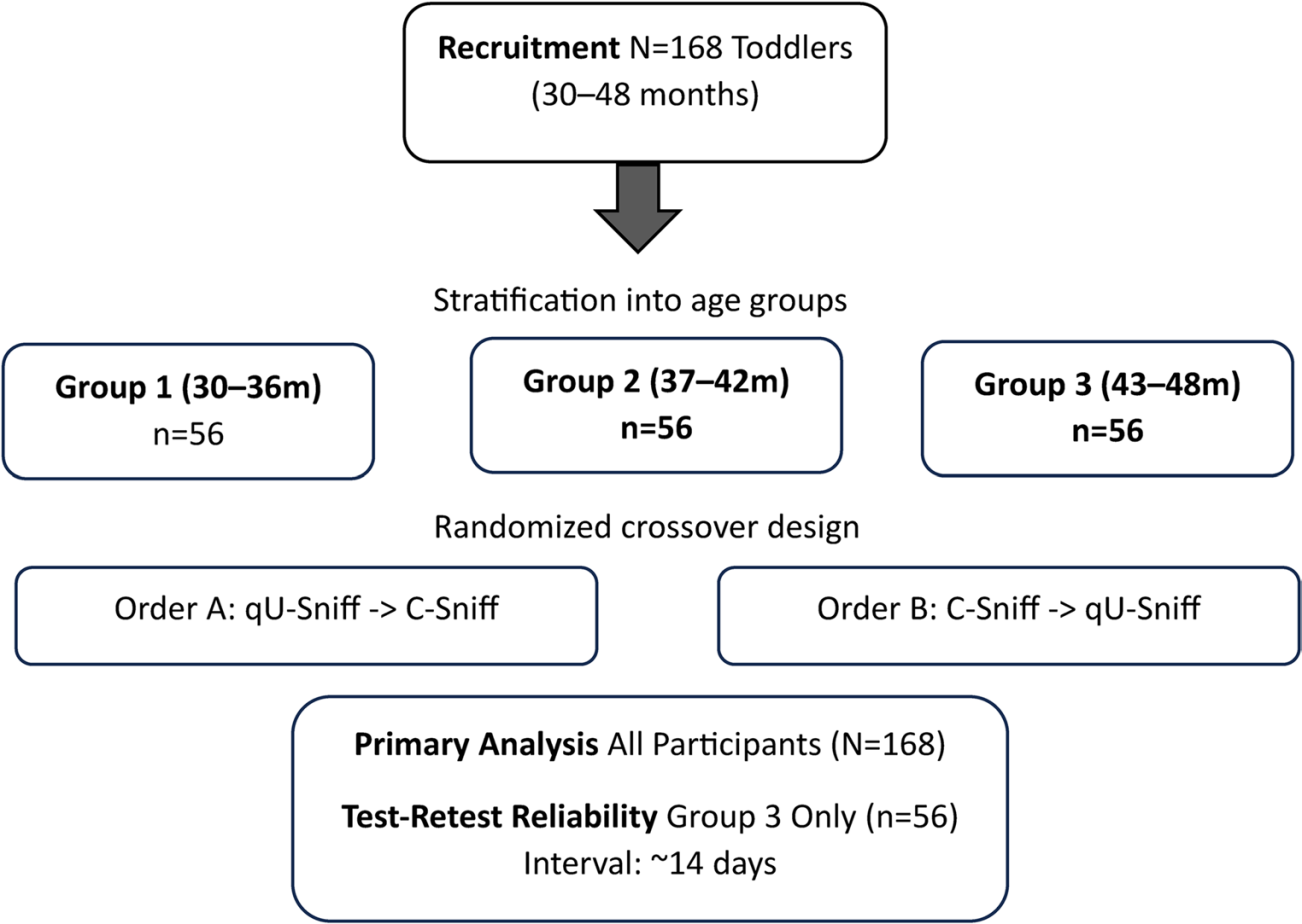
Study Design Flow Chart. This diagram illustrates the recruitment of 168 toddlers and their stratification into three age groups (Group 1: 30–36 months; Group 2: 37–42 months; Group 3: 43–48 months). Each group followed a randomized crossover design to compare the qU-Sniff and C-Sniff tests. Test-retest reliability was assessed in the oldest age group (*n* = 56) after an interval of approximately 14 days

The 168 subjects in the main analysis group were randomly assigned to two groups to control for a possible order effect. Group (1) first performed the qU-Sniff and then the C-Sniff. Group (2) performed these in reverse order. The study was conducted in the presence of the child’s legal guardians at the kindergarten, who completed a short questionnaire in advance about the child’s previous illnesses and development. After completing the tests, the children were asked which of the two tests they preferred. The subjects in the retest group, 43–48 months, were retested after an average interval of 13.8 days (median: 14 days) to determine test reliability (Fig. 1).

**Fig. 2:**
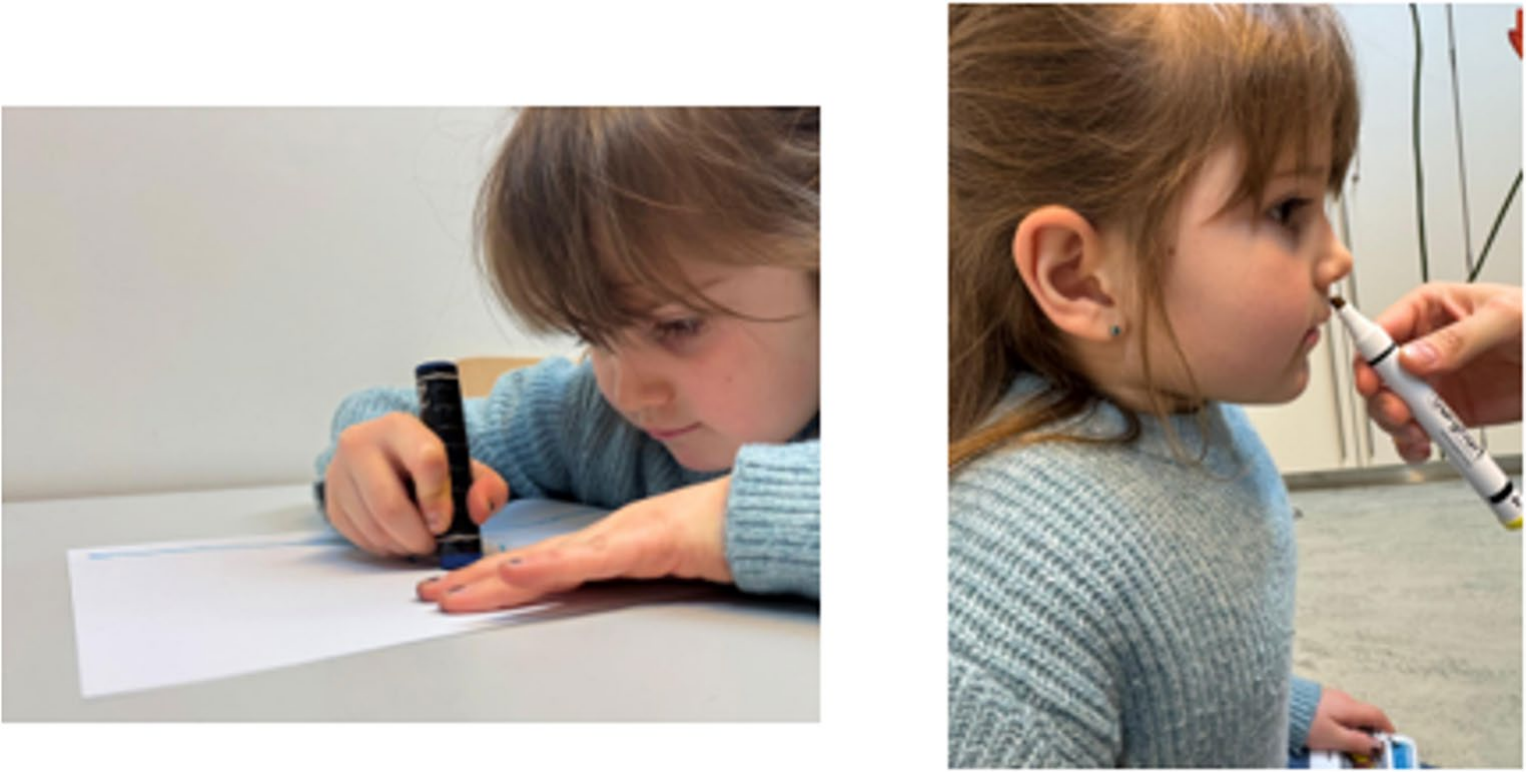
Photos of a girl using the C-Sniff (left) and qU-Sniff test (right)

Olfactory testing was performed using two methods for odor identification: The qU-Sniff: A shortened version of the established U-Sniff set was used, consisting of five specific Sniffin‘ Sticks that had to be matched to picture cards [5]. The test utilized a four-alternative forced-choice (4-AFC) design, with one correct picture and three distractors. The odors were held approximately 2 cm below the child’s nose. The crayon sniff (C-Sniff): As a novel method, blue wax crayons with the same five odors (coffee, peach, flower, fish, and onion) as in the qU-Sniff were used (Noar, Sao Paulo, Brazil). The children were instructed to draw with the crayons to release the odor and then identify it using picture cards, again in a four-alternative forced-choice design (Fig. 2).

The sample size was determined based on a priori power analysis (G*Power): to detect a medium effect size (d = 0.6) with a power of 0.9 and α = 0.05 for t-tests, a minimum of 32 subjects per age group was required. To further increase validity and account for potential dropouts, we aimed for 56 subjects per age group, resulting in the final sample of *N* = 168.

All statistical analyses were performed using the R environment (version 4.5.0; R Core Team, 2025). Data processing and analysis were conducted within the RStudio integrated development environment (version 2025.05.1; RStudio Team, 2025). The significance level was set at α = 0.05. Descriptive analysis: Numerical variables (e.g., age, test results) were summarized using mean, median, and standard deviation. Categorical variables (e.g., gender, dropout rate) were described using frequencies and percentages. Normality of data distribution was assessed using the Shapiro-Wilk test and visual inspection of Q-Q plots. For the identification scores, the Shapiro-Wilk test indicated a significant deviation from normality (*p* < 0.001). Consequently, non-parametric tests (Wilcoxon Signed-Rank Test, Kruskal-Wallis Test) were primarily used. The McNemar test was used to compare dropout rates between qU-Sniff and C-Sniff. The relationship between dropout rate and age group was analyzed using a chi-square test. The Wilcoxon Signed-Rank Test was employed for the paired comparison of identification scores, while a paired t-test was used to assess significant differences in processing times. The Mann-Whitney U test and Welch’s t-test were used to compare means and distributions between the two orders and gender groups. Pearson and Spearman correlations were calculated to examine the relationship between kindergarten hours and test results. Test-retest reliability was determined by correlating the test and retest results and by calculating the Intraclass Correlation Coefficient (ICC).

## Results

The main analysis was based on results from 168 participants. The oldest age group was retested for reliability. The sample included children aged 30 to 48 months. The 168 subjects in the main analysis were evenly distributed across three age groups (30 to 36 months, 37 to 42 months, 43 to 48 months) and gender. In accordance with the study protocol, participants were randomly assigned to one of two study groups to vary the order of the tests (qU-Sniff, C-Sniff).

Across all age groups, a mean qU-Sniff score of M = 2.92 (SD = 1.57) and a mean C-Sniff score of M = 2.77 (SD = 1.60) were obtained. The subsequent analysis of identification performance across the three defined age groups revealed a highly significant effect of age on both test scores. Kruskal-Wallis tests showed a statistically significant difference between the groups for both the qU-Sniff Score (H = 24.86, *p* < 0.001) and the C-Sniff Score (H = 24.53, *p* < 0.001). Post-hoc pairwise comparisons using Wilcoxon rank-sum tests with Bonferroni correction further specified these differences: For the qU-Sniff, the youngest group (Group 1, 30–36 months) performed significantly worse than Group 2 (37–42 months; *p* = 0.003) and Group 3 (43–48 months; *p* < 0.001). No significant difference was found between the two older groups (*p* = 0.65).

Regarding the C-Sniff, significant differences were observed between Group 1 and Group 3 (*p* < 0.001) as well as between Group 2 and Group 3 (*p* = 0.035). The difference between Group 1 and Group 2 showed a trend but did not reach statistical significance after correction (*p* = 0.065).

The results indicated a clear age-dependent improvement in olfactory performance. For the qU-Sniff task, the mean scores increased consistently with age: M = 2.07 (SD = 1.62) was recorded for group 30 to 36 months, which rose to M = 3.11 (SD = 1.52) for group 37 to 42 months, and finally peaked at M = 3.57 (SD = 1.31) in the oldest group 43 to 48 months. A similar pattern was observed for the C-Sniff score, starting at a mean of M = 2.02 (SD = 1.61) for group 30 to 36 months, increasing to M = 2.75 (SD = 1.55) in group 37 to 42 months, and reaching M = 3.55 (SD = 1.40) in the oldest group 43 to 48 months. The oldest group achieved the highest scores in both tests.

**Fig. 3:**
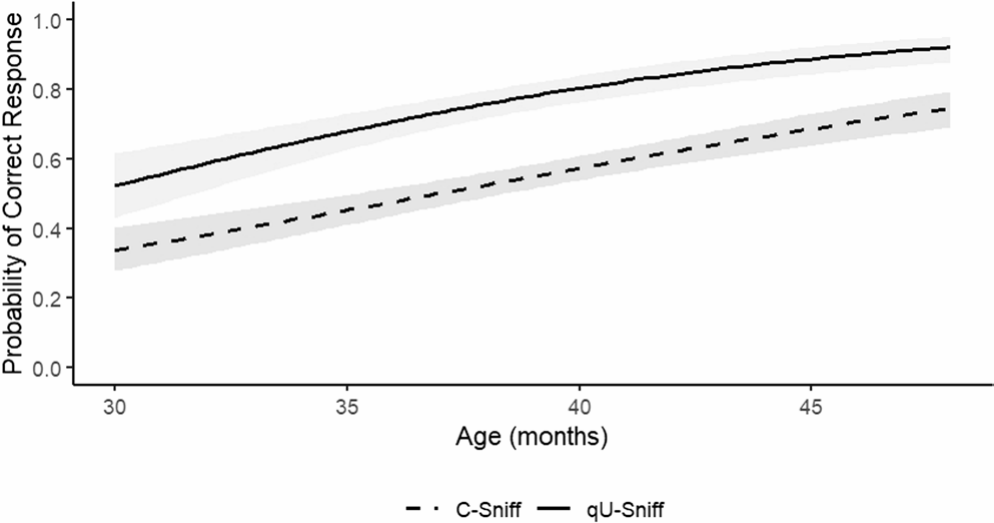
Logistic regression analysis of odor identification probability by age. The dashed line represents the C-Sniff and the solid line represents the qU-Sniff. The shaded areas indicate the 95% confidence intervals. For both tests, age (as a continuous variable in months) was a highly significant predictor of performance (both *p* < 0.001), showing a steady increase in identification probability during the fourth year of life

To further validate the age-dependency, a logistic regression analysis was conducted using age in months as a continuous variable. The analysis confirmed a highly significant impact of age on odor identification performance for both tests. For each additional month of age, the odds of a correct odor identification increased by 14% for the qU-Sniff (OR = 1.14, 95% CI [1.09–1.19], *p* < 0.001) and by 10% for the C-Sniff (OR = 1.10, 95% CI [1.07–1.13], *p* < 0.001). These results demonstrate a continuous and progressive development of olfactory skills during the fourth year of life (Fig. 3).

Comparison of tests in terms of feasibility and acceptance: There was no significant difference in the overall discontinuation rate between the qU-Sniff (7.7%, *n* = 13) and the C-Sniff (8.3%, *n* = 14) (McNemar test, *p* = 1). However, discontinuation rates varied significantly by age group. For the qU-Sniff, the discontinuation rate in the youngest age group (group 30–36 months) was 19.6% (*n* = 11), compared to 1.8% (*n* = 1) in the two older groups (30 to 36 and 37 to 42 months) (chi-square test, *p* < 0.001). A similar pattern was seen with the C-Sniff, where group 30 to 36 months also had significantly higher discontinuation rates at 17.9% (*n* = 10) compared to the older age groups (chi-square test, *p* = 0.005).

Of the 155 participants who indicated a preference regarding the two tests, 51.6% (*n* = 80) chose the C-Sniff and 48.4% (*n* = 75) chose the qU-Sniff. This distribution did not differ significantly from a 50/50 distribution (chi-square test, *p* = 0.688). There was also no significant association between preference and age group (Fisher’s exact test, *p* = 0.959).

**Fig. 4:**
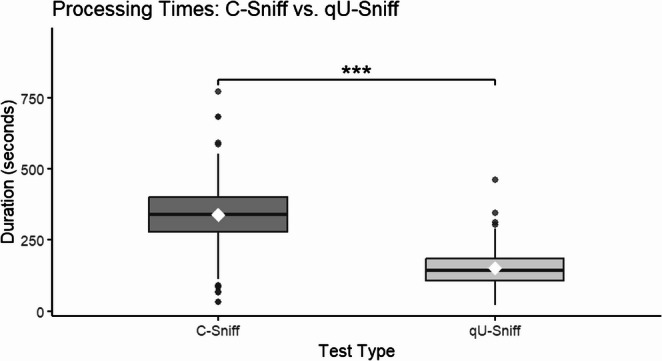
Statistical comparison of administration duration. The boxplots illustrate the processing times for the C-Sniff (5 items) and the qU-Sniff (3 items). The horizontal line represents the median, while the white diamond indicates the mean value. The significance bracket (* *p* < 0.001)** highlights the substantial difference in efficiency between the two tests. While individual variability exists (as indicated by the whiskers), the qU-Sniff is significantly faster to administer, making it more suitable for clinical settings with limited time resources

Comparison of the tests in terms of processing time and reliability: The processing time for the C-Sniff was significantly longer than for the qU-Sniff, with a mean of 352.4 s (median: 350.5 s) compared to a mean of 160.5 s (median: 151 s) (paired t-test, *p* < 0.001) (Fig. 4). A significantly negative mean difference of −191.9 s was observed (95% CI: −207.04 to −176.77). The order of the tests had a significant effect on processing times, but not on the identification scores. Participants who started with the qU-Sniff (group 30 to 36 months) took an average of 173.4 s, while those who did it second (group 37 to 42 months) took an average of 147.6 s (Mann-Whitney U test, *p* = 0.007). A similar effect was observed for the C-Sniff, which took less time when performed as the second test (mean 327.4 s) compared to when performed at the beginning of the study (mean 377.4 s) (Mann-Whitney U test, *p* < 0.001).

The retest analysis was performed on 56 participants from the oldest age group. The mean time interval was 13.75 days. Both tests showed moderate, significant test-retest reliability: qU-Sniff: Pearson correlation *r* = 0.526 (*p* < 0.001), ICC = 0.52, and C-Sniff: Pearson correlation *r* = 0.548 (*p* < 0.001), ICC = 0.539.

Influence of daycare hours and gender: There was neither a significant correlation between the number of daycare hours and the test results (qU-Sniff: *p* = 0.578; C-Sniff: *p* = 0.895) nor a significant difference in the test results between the genders (qU-Sniff: *p* = 0.535; C-Sniff: *p* = 0.082).

## Discussion

The presented study gives important knowledge about the feasibility and the test-retest reliability of two odor identification tests in the challenging age range between 30 and 48 months. The results show that already presented short U-Sniff (qU-Sniff) as well as the new Crayon-Sniff (C-Sniff) have a moderate reliability, which demonstrates their suitability in this age cohort.

As in previous studies, the odor identification ability increased with age, although this has not been tested in age groups as young as those in the present study [2, 6, 7]. The observed increase in identification performance with age may be influenced by the parallel development of verbal abilities and semantic memory in toddlers [2, 8, 9]. Although formal language testing was not part of this study, the interplay between linguistic maturation and olfactory naming is a relevant factor that should be addressed in future investigations.

The significant difference between the two tests in practical use is the time efficiency. The C-Sniff requires more than twice the time as the qU-Sniff, nearly six minutes in comparison to less than three minutes as a mean. Another advantage of qU-Sniff is that it is already validated. It should be noted that the test-retest reliability for the qU-Sniff was slightly lower than in the original publication presenting this test, which can be explained by the young age group in this study [5].

The development of the C-Sniff is based on the hope to receive a more active, playful approach through drawing and enhance the compliance and acceptance in kindergarten age. While the C-Sniff had a slightly higher subjective preference among the participants, this was not displayed in the dropout rate in the study. The longer duration of the C-Sniff, therefore, is a clear disadvantage. In a clinical and study-based context, the concentration span of toddlers is a limiting factor. In clinical and large-scale study contexts, where concentration and attention are limiting factors in toddlers, the qU-Sniff is more efficient because it requires only half the time.

In both variants of testing, a moderate test-retest reliability (ICC-qU-Sniff​=0.52; ICC-C-Sniff​=0.539) in the context of the age group 43 to 48 months has been found, which can be rated as acceptable. In toddlers, the natural variability of the ability to concentrate, the fluctuating interest, and the requirement of verbal abilities to less robust psychometric scores than in older children or adults. The comparable reliability of both tests suggests that both methods are fundamentally capable of measuring olfactory identification performance, but that the results can be influenced by factors specific to the day in question. The significantly higher dropout rate in both tests in younger age groups (30 to 36 months) displays the boundaries of the current identification tests. This underscores the need to either develop even more abbreviated procedures for the youngest toddlers or to switch to nonverbal test paradigms for assessing pure odor perception, as the cognitive and linguistic demands of identification tests in this group may mask actual olfactory function.

Previous tests have primarily attempted to apply established odor testing paradigms to younger age groups without further adaptation. Examples include the original U-Sniff. There is a publication on this for children aged three to six. The test-retest reliability for four-year-olds corresponds to that in our study, which is not surprising given that the qU-Sniff was developed as a short screening test based on this test [10]. As part of the development of the NIH Toolbox for recording neurobehavioral measurements to rapidly assess cognitive, emotional, sensory, and motor functions, an assessment of odor identification ability was implemented. A test for identifying six different odors was developed for children aged 3 to 9 years using a four-alternative forced-choice (4-AFC) method [11, 12]. However, a subsequent study revealed that it was challenging to administer the test to children aged three to four [12]. A test to identify odors was developed in Poland for children from Eastern Europe. The full version of the test comprises 21 odors, which had to be identified using a 3-AFC method with pictures. Additionally, a shorter screening test comprising six odors was introduced [13]. However, there is no validation or normative data for this test.

Currently, no validated normative values exist for olfactory identification in children aged 30 to 48 months. While clinical screening in older children typically expects near-perfect scores (5/5), our data suggests that for toddlers, the statistical chance level (1.25/5) is the more appropriate benchmark for interpreting early results. The transition from near-chance performance in the youngest group (mean score 2.0) to more robust identification in older toddlers (mean score 3.5) underscores that ‘failing’ a test at age 2.5 should be interpreted with caution. These findings highlight the need for age-specific norms that account for the steep developmental curve observed in this study. The data available for this age group is extremely limited. It should be noted that the tools presented here, qU-Sniff and C-Sniff, can only be used for brief screenings. If any unusual results occur, it is advisable to repeat the test and, if necessary, carry out further testing over time [14].

The significant order effect on processing time is methodologically interesting: both tests were completed faster when they were performed as the second test. This finding suggests a learning effect, whereby the children were familiar with the general concept of odor presentation, identification, and interaction with the examiners after the first test. Future studies must take this effect into account in their statistical analysis and, if possible, control for it through appropriate randomization.

A limitation of the present study is that test-retest reliability was exclusively assessed in the oldest subgroup (42–48 months). In younger toddlers, factors such as fluctuating attention, rapid fatigue, and significant learning effects between administrations make formal reliability testing challenging. Therefore, our reliability findings should be interpreted as a ‘best-case’ stability measure. Future studies with larger cohorts should aim to implement staggered re-testing intervals to better account for the developmental volatility in children under three years of age. Beyond the age-specific reliability analysis, other limitations of this study warrant consideration. First, the sample was recruited primarily from local daycare centers, which may limit the generalizability of the findings to a more diverse socio-economic or cross-cultural population. Second, while the sample size was sufficient to detect significant developmental trends, larger cohorts would be required to establish definitive normative centiles. Furthermore, olfactory performance in toddlers is inherently susceptible to non-sensory factors, such as daily fluctuations in motivation, hunger, or minor respiratory congestion not clinically apparent during screening. Finally, the cross-sectional nature of the study provides a ‘snapshot’ of development; longitudinal data would be ideal to more accurately track individual maturation trajectories in odor identification skills.

## Conclusion

Both the qU-Sniff and C-Sniff are moderately reliable tools for assessing olfaction in toddlers. However, the playful C-Sniff is significantly more time-consuming. Future studies should consider the age-related challenges and the significant sequence effects on test duration.

## Data Availability

The data underlying this article cannot be shared publicly due to the data protection law of the country of origin.
